# Maximizing the Spread of Influence via Generalized Degree Discount

**DOI:** 10.1371/journal.pone.0164393

**Published:** 2016-10-12

**Authors:** Xiaojie Wang, Xue Zhang, Chengli Zhao, Dongyun Yi

**Affiliations:** 1 College of Science, National University of Defense Technology, Changsha, Hunan, China; 2 State key laboratory of High Performance Computing, National University of Defense Technology, Changsha, Hunan, China; Semmelweis University, HUNGARY

## Abstract

It is a crucial and fundamental issue to identify a small subset of influential spreaders that can control the spreading process in networks. In previous studies, a degree-based heuristic called *DegreeDiscount* has been shown to effectively identify multiple influential spreaders and has severed as a benchmark method. However, the basic assumption of *DegreeDiscount* is not adequate, because it treats all the nodes equally without any differences. To consider a general situation in real world networks, a novel heuristic method named *GeneralizedDegreeDiscount* is proposed in this paper as an effective extension of original method. In our method, the status of a node is defined as a probability of not being influenced by any of its neighbors, and an index generalized discounted degree of one node is presented to measure the expected number of nodes it can influence. Then the spreaders are selected sequentially upon its generalized discounted degree in current network. Empirical experiments are conducted on four real networks, and the results show that the spreaders identified by our approach are more influential than several benchmark methods. Finally, we analyze the relationship between our method and three common degree-based methods.

## Introduction

In complex networks, models and methods for propagation behavior are always of great theoretical and practical importance. Consider a scenario in advertising: a small IT company develops a cool online application and it wants to let more people know their product. However, the funds for advertising are limited. An economical way to advertise is to deliver the product to a small group of initial users(or spreaders) who are willing to advertise the product by word of mouth. This is often referred to as influence maximization. Theoretically, influence maximization in networks is a specific problem about how to effectively identify a small subset of nodes and maximize their spreading influence. Although much work has been done on measuring the influence of a single node [1–6], methods that can effectively identify multiple influential spreaders are still lacking.

The pioneers of this research area are Domingos and Richardson [7, 8] who studied influence maximization as an algorithmic problem and developed a probabilistic method. Kempe, Kleinberg and Tardos [9] also made a significant contribution to this field. They showed that the problem is an NP-hard discrete optimization problem, and proposed a greedy strategy to select the spreaders that could achieve an approximation guarantee of 63%. Unfortunately, their greedy method encountered a serious drawback in computing efficiency, which limited its wide usage in large-scale networks. Leskovec *et al.* [10] demonstrated that many realistic influence maximization problems exhibit a property of “submodularity”, and they proposed a *Cost-Effective Lazy Forward(CELF)* method to improve the efficiency of the greedy method. Narayanam *et al.* [11] analyzed the Shapley value concept from cooperative game theory and proposed *ShaPley value-based Influential Nodes(SPIN)*. Zhao *et al.* [12] attempted to find effective multiple spreaders by generalizing the idea of the coloring problem in graph theory to complex networks. He *et al.* [13] suggested a novel method to identify multiple spreaders from communities in a balanced way. Zhang *et al.* [14] presented an iterative method named VoteRank to identify a set of decentralized spreaders. Chen *et al.* [15] decomposed the local topological structure of nodes and proposed a *DegreeDiscount* heuristic. Numerical experiments showed that *DegreeDiscount* could nearly match the performance of the greedy method, while the computational complexity of the former one was quite low. However, all nodes were treated equally in *DegreeDiscount*, which was a little oversimplified and might reduce the performance of the algorithm.

In this paper, we depict the status of nodes more concisely as a probabilistic score, and propose the *GeneralizedDegreeDiscount* heuristic. We discuss the computational efficiency of our method and demonstrate that the complexity is linearly correlated with the network scale, which makes our method efficient and scalable to large-scale networks. Experiments are performed on several real networks, and the results show that our method can outperform some centrality-based methods.

## Materials and Methods

### Intuition and theory

*Degree* is a basic centrality index in the research area of complex networks. It is well known that a node with a higher degree can influence more nodes than a node with a lower degree. Some researches in sociology have shown that selecting nodes with the highest degree as spreaders can result in better spreading influence than many other methods. However, in some recent studies, the authors argued that nodes with the highest degree might not always be the most influential ones. Though the effectiveness of the *Degree* is questionable, the low computational complexity of this strategy results in its widespread use in many business fields. In this section, we try to enhance the performance of this method by using several heuristic strategies.

Let node *v* be a neighbor of node *u*. Suppose *u* has been selected as a spreader. When considering the selection of *v* as a new spreader, one should formulate a method for calculating the contribution of edge $\bar{uv}$ to the degree of node *v*. It cannot be counted as 1, as has been done previously. As *u* has been selected as a spreader: (i) it is no longer necessary for *v* to influence *u*. (ii) *u* may also influence *v* with some probability, which further weakens the potential influence of *v*. Based on these considerations, we explore several heuristic strategies, in which the spreaders are selected one by one.

#### Degree-based heuristics

*DegreeDistance* [16] takes a naive approach to avoid the relative influence between adjacent nodes. For example, if a node has been selected as a spreader, we can ignore its neighbors and consider other nodes. *DegreeDistance* defines a candidate set *C* and a distance threshold *d*_*td*_. At first, all the nodes are in the candidate set *C*. In each round, a node *v* with maximum degree in *C* is selected as a spreader, and the nodes within a distance *d*_*td*_ to *v* are removed from *C*. The procedure ends when all the spreaders have been selected.

In Ref [15], Chen *et al.* proposed two degree-based heuristics, *SingleDiscount* and *DegreeDiscount*. *SingleDiscount* considers a simple adaptive strategy. In each round, a node *u* with the maximum degree is selected as a spreader. Then, for each *v* ∈ Γ(*u*), we do not count $\bar{uv}$ when calculating its degree. In other words, the degree of *v* will be discounted by 1. This type of degree is named as discount degree, and is denoted by *sd*_*v*_

$$\begin{array}{c} sd_{v}=d_{v}-t_{v} \end{array}$$ (1)

where *d*_*v*_ denotes the original degree of *v*, and *t*_*v*_ denotes the number of *v*’s neighbors who have already been selected as spreaders.

*DegreeDiscount* is specifically designed for the independent cascade model. For a specific spreading probability *p*, *DegreeDiscount* attempts to conduct a deeper analysis of the local structure of the nodes. Suppose that we want to calculate the potential spreading ability of node *v*. Let the spreading probability be *p*. When *p* is small, the multi-hop neighbors of *v* can be ignored, and only the nearest neighbors are counted toward the degree. Let *u* ∈ Γ(*v*) be a spreader neighbor of *v*. Obviously, the probability that *v* is directly influenced by *u* is *p*. As a result, *u* will not only contribute nothing to *v*, but also weakens the spreading ability of *v*.

When calculating the potential spreading ability of *v*, *DegreeDiscount* ignores the differences of *v*’s neighbors. As all the neighbors are treated equally, the diagram of *v* and its neighbors Γ(*v*) can be mapped into a star-like subgraph structure. Let *Star*(*v*) be the subgraph considered here, and let the edges in the subgraph be the edges incident to *v*. Let *d*_*v*_ be the degree of node *v*, *t*_*v*_ be the number of spreader neighbors of *v*, and *p* be the spreading probability.

As the candidate node *v* has *t*_*v*_ spreader neighbors, the probability that *v* is influenced by these neighbors is $1-{\left(1-p\right)}^{t_{v}}$. In this situation, selecting *v* as a new spreader may not bring any additional influence. In the opposite situation, selecting *v* will contribute to the spreading process by *v* itself and its normal neighbors. The former term can influence 1 node(*v* itself), and the latter can influence *d*_*v*_ − *t*_*v*_ nodes(normal neighbors) with probability *p*. Together, the expected number of nodes influenced by *v* is

$$\begin{array}{c} {\left(1-p\right)}^{t_{v}}\left(1+\left(d_{v}-t_{v}\right)p\right) \end{array}$$ (2)

Under the first order of Taylor expansion, when *p* is small, the left term can be approximated by 1 − *t*_*v*_ *p* + *o*(*t*_*v*_ *p*). After further simplification, the whole equation becomes

$$\begin{array}{c} 1+\left(d_{v}-2t_{v}-\left(d_{v}-t_{v}\right)t_{v}p\right)p+o\left(t_{v}p\right) \end{array}$$ (3)

Then, the discounted degree of *v* can be defined as

$$\begin{array}{c} dd_{v}=d_{v}-2t_{v}-\left(d_{v}-t_{v}\right)t_{v}p \end{array}$$ (4)

**Fig 1:**
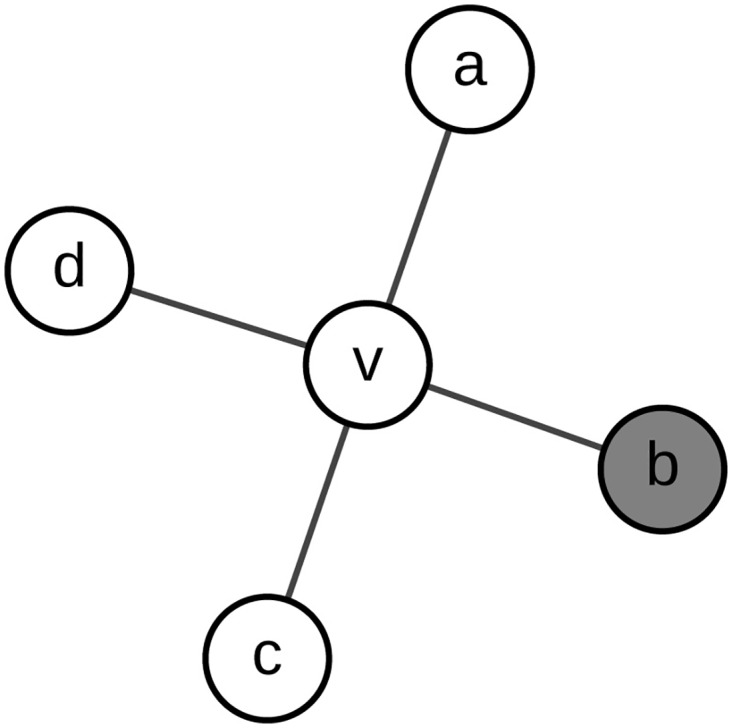
The diagram of *DegreeDiscount*. Nodes filled by gray denote the selected spreaders, and others denote normal nodes.

Note that in the original equation Eq (2), *dd*_*v*_ is always non-negative. However, in the simplified form Eq (4), *dd*_*v*_ may be negative with some special parameters. In this situation, we manually set *dd*_*v*_ to be 0. Fig 1 depicts the local topology considered by *DegreeDiscount*. In this toy model, *d*_*v*_ = 4, *t*_*v*_ = 1, and

$$\begin{array}{c} dd_{v}=4-2\times 1-\left(4-1\right)\times 1p=2-3p \end{array}$$ (5)

#### Generalized Degree Discount

As *DegreeDiscount* models all the neighbors in Γ(*v*) equally, it ignores the differences among them. Take an extreme case as example, let *s*, *t* ∈ Γ(*v*) be two normal neighbors of node *v*. If all the neighbors of *s* itself are spreaders and all the neighbors of *t* itself are normal nodes, they should not be treated equally. Obviously, the probability that *s* is influenced by its own neighbors is far larger than *t*. When calculating the potential contribution of *s*, *t* towards *v*, the latter one should be given more weight. To make the original model more precisely, we propose the *GeneralizedDegreeDiscount*.

Similar to the analysis in *DegreeDiscount*, the probability that node *v* is not influenced by its spreader neighbors is ${\left(1-p\right)}^{t_{v}}$. If *v* is not influenced by any of those neighbors, selecting *v* will enhance the total influence by *v* itself and its *d*_*v*_ − *t*_*v*_ normal neighbors. For any normal neighbor *w* ∈ Γ(*v*), the probability that *w* is not influenced by its own neighbors is also ${\left(1-p\right)}^{t_{w}}$. In other words, *w* will bring additional influence ${\left(1-p\right)}^{t_{w}}$ to *v* with probability *p*. Together, the expected number of nodes that will be influenced by *v* is

$$\begin{array}{c} {\left(1-p\right)}^{t_{v}}\left(1+\sum_{d_{v}-t_{v}}{\left(1-p\right)}^{t_{w}}p\right) \end{array}$$ (6)

where the summation is over all *d*_*v*_ − *t*_*v*_ normal neighbors of node *v*.

Departing from the conduction in *DegreeDiscount*, here we consider the second-order Taylor expansion for the left term and the first-order expansion for the right term:

$$\begin{array}{c} \left(1-t_{v}p+\frac{1}{2}t_{v}\left(t_{v}-1\right)p^{2}+o\left({\left(t_{v}p\right)}^{2}\right)\right)\left(1+\sum_{d_{v}-t_{v}}\left(1-t_{w}p+o\left(t_{w}p\right)p\right)\right) \end{array}$$ (7)

After further simplification, the equation becomes

$$\begin{array}{c} 1+\left(d_{v}-2t_{v}-\left(d_{v}-t_{v}\right)t_{v}p+\frac{1}{2}t_{v}\left(t_{v}-1\right)p-\sum_{d_{v}-t_{v}}t_{s}p\right)p+o\left(t_{v}^{2}p^{2}\right)+\sum_{d_{v}-t_{v}}o\left(t_{w}p^{2}\right) \end{array}$$ (8)

Then, the generalized discounted degree of *v* can be defined as

$$\begin{array}{c} gdd_{v}=d_{v}-2t_{v}-\left(d_{v}-t_{v}\right)t_{v}p+\frac{1}{2}t_{v}\left(t_{v}-1\right)p-\sum_{d_{v}-t_{v}}t_{w}p \end{array}$$ (9)

**Fig 2:**
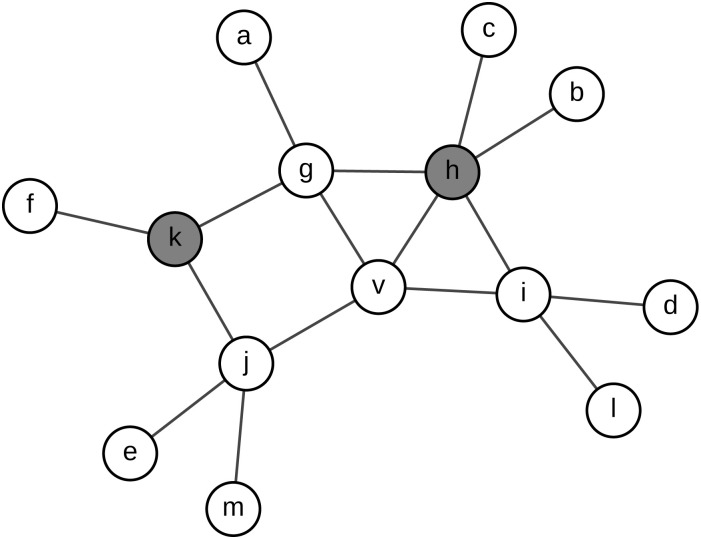
The diagram of *GeneralizedDegreeDiscount*. Nodes filled by gray denote the selected spreaders, and others denote normal nodes.

Similar to the situation in *DegreeDiscount*, the simplified equation of *gdd*_*v*_ may also be less than zero. In our real implementations, we set *gdd*_*v*_ = 0 in this situation. Fig 2 depicts the local topology considered by *GeneralizedDegreeDiscount*. In this toy model, *d*_*v*_ = 4 and *t*_*v*_ = 1. Note that the summation is over all normal neighbors, i.e., {*g*, *i*, *j*} in this figure. As *t*_*g*_ = 2, *t*_*i*_ = 1 and *t*_*j*_ = 1, the generalized discounted degree of *v* is

$$\begin{array}{c} gdd_{v}=4-2\times 1-\left(4-1\right)\times 1p+\frac{1}{2}\times 1\times \left(1-1\right)p-\left(2+1+1\right)p=2-7p \end{array}$$ (10)

Compared to the formulation of *DegreeDiscount*(Eq (4)), the formulation of *GeneralizedDegreeDiscount*(Eq (9)) adds the last two terms. As the consideration of the latter one is deeper than the former one, *GeneralizedDegreeDiscount* should be more effective than *DegreeDiscount*. However, the difference between them is not so significant in real situations. In reality, a small fraction of spreaders must be selected and their influences broadcast with low probability. Because the number of spreaders is not large, usually *t*_*v*_ ≪ *d*_*v*_ for all nodes. Thus, in Eq (9), the fourth and fifth terms are smaller than the third term, which makes *GeneralizedDegreeDiscount* just similar to *DegreeDiscount*. In the Results section, we will compare the two methods numerically.

### Computational efficiency

The *GeneralizedDegreeDiscount* is implemented in Algorithm 1. If we want to select *l* spreaders, the algorithm must run for *l* rounds. Let *N* be the number of nodes and 〈*k*〉 be the average degree. In each round, the selection scheme costs *O*(*N*), the neighbor finding scheme costs *O*(〈*k*〉^2^), and for each of those neighbors, the updating process costs *O*(〈*k*〉). Then, the total time cost of the algorithm is *O*(*l*(*N* + 〈*k*〉^2^ + 〈*k*〉^2^ ⋅ 〈*k*〉)) ≈ *O*(*l*(*N* + 〈*k*〉^3^)). In many networks, the average degree is far less than the number of nodes: 〈*k*〉 ≪ *N*. Thus the time cost of *GeneralizedDegreeDiscount* will be nearly *O*(*lN*), which is just linearly correlated with the scale of the network.

**Algorithm 1** GeneralizedDegreeDiscount(*G*, *l*, *p*)

Initialize *S* = ∅

**for all** *v* ∈ *V* **do**

 *gdd*_*v*_ = *d*_*v*_

 *t*_*v*_ = 0

**end for**

// iteratively select *l* spreaders

**for** *i* = 1 *to* *l* **do**

 // select the node with the maximum generalized discount degree *gdd*_*v*_

 select *u* = *arg* *max*_*v*_ {*gdd*_*v*_|*v* ∈ *V*∖*S*}

 *S* = *S* ∪ {*u*}

 *NB* = ∅

 // find the nearest and next nearest neighbors of *u* and update *t*_*v*_ for *v* ∈ Γ(*u*)

 **for all** *v* ∈ Γ(*u*) **do**

  *NB* = *NB* ∪ {*v*}

  *t*_*v*_ = *t*_*v*_ + 1

  **for all** *w* ∈ Γ(*v*) **do**

   *NB* = *NB* ∪ {*w*}

  **end for**

 **end for**

 // update *gdd*_*v*_ for all *v* ∈ *NB*

 **for all** *v* ∈ *NB* **do**

  *sum*_*tw*_ = 0

  **for all** *w* ∈ Γ(*v*) **do**

   **if** *w* ∉ *S* **then**

    *sum*_*tw*_ = *sum*_*tw*_ + *t*_*w*_

   **end if**

  **end for**

$gdd_{v}=d_{v}-2t_{v}-\left(d_{v}-t_{v}\right)t_{v}p+\frac{1}{2}t_{v}\left(t_{v}-1\right)p-sum_{tw}p$

  **if** *gdd*_*v*_ < 0 **then**

   *gdd*_*v*_ = 0

  **end if**

 **end for**

**end for**

**return** *S*

## Results and Discussion

To evaluate the performance, we simulate the experiments using the Susceptible-Infected-Recovered(SIR) model. The SIR model was originally proposed as a model of the dynamics of the spread of disease. Due to the similarities between epidemic transmission and the spread of information, we use SIR to measure the spreading influence of individual nodes. In the SIR model, a node may assume one of three states(susceptible, infected and recovered). Specifically, susceptible individuals *S* in the model is analogous to individuals who are not aware of the information. Infected individuals *I* can be analogous to information carriers who are willing to spread information to their neighbors. Recovered individuals *R* are those who had previously received the information but later lost interest. To better simulate the real-world spreading process, we use the SIR model with limited contact [17]. At each time step, each infected node will randomly select a neighbor to contact, and will transmit the disease to its neighbor with probability *p* if the neighbor is susceptible. After the transmission process, the infected node will become a recovered node with probability *q*. The effective spreading rate *λ* is defined as *p*/*q*. When there are no infected nodes, the process stops, and we use the fraction of recovered nodes to measure the spreading influence.

### Data Description

To evaluate the influences of different groups of spreaders selected by various methods, we conduct the experiments on the following four networks from different fields.

1. Enron [18]: An email communication network which covers all the email communication within Enron Corporation. Nodes in the network are email addresses and edges represent the email communications among them.
2. Cond-mat [19]: A collaboration network of scientists posting preprints to the condensed matter archive at arxiv.org between January 1, 1995 and March 31, 2005. Nodes in the network represent the scientists and edges represent the collaborations among them.
3. Gnutella [20]: A snapshot of the Gnutella peer-to-peer file sharing network at August 31 2002. Nodes represent hosts in the Gnutella network topology and edges represent connections between the Gnutella hosts.
4. Epinions [21]: A who-trust-whom online social network of the general consumer review site Epinions.com. All the trust relationships interact and form the Web of Trust, which is then combined with review ratings to determine which reviews are shown to the users.

For simplicity, we treat all the networks as undirected and unweighted, and discard self-loops and multiple links. Only the largest connected component of each network is considered. A Brief overview of the networks is shown in Table 1.

**Table 1:** The basic topological features of four real networks. *N* and *M* are the numbers of nodes and edges. 〈*k*〉 is the average degree. *d*_*max*_ denotes the network diameter and 〈*d*〉 denotes the average shortest path length. *r* and *cc* are the assortative coefficient and clustering coefficient, respectively.

*network*	*N*	*M*	〈*k*〉	*d*_*max*_	〈*d*〉	*r*	*cc*
Enron	33696	180811	10.732	13	4.025	-0.116	0.085
Cond-mat	36458	171736	9.421	18	5.499	0.177	0.243
Gnutella	62561	147878	4.727	11	5.936	-0.093	0.004
Epinions	75877	405739	10.695	15	4.308	-0.041	0.066

### Benchmark methods

In complex networks, many centrality indexes have been defined to measure the importance of nodes and links. It is believed that nodes with higher centrality are more influential than common nodes. Accordingly, one naive solution for the influence maximization problem is to select the *top* − *l* nodes with the highest centrality indexes. In this paper, we use centrality-based methods as the benchmark methods.

*Degree* is a basic local centrality index for nodes. The higher degree a node has, the more important it is. In a social network, a person with more followers or friends is likely to have a larger influence.

*Betweenness* [22, 23] measures the extent to which a node is located on the shortest paths between pairs of nodes in networks.

$$\begin{array}{c} C_{B}\left(v\right)=\sum_{s\neq v\neq t}\frac{\sigma_{st}\left(v\right)}{\sigma_{st}} \end{array}$$ (11)

where *σ*_*st*_ denotes the number of shortest paths between a pair (*s*, *t*) of nodes, and *σ*_*st*_(*v*) denotes the number of shortest paths between any pair of nodes that pass through *v*.

*Closeness* [24] is an evaluation of the geometric location of nodes.

$$\begin{array}{c} C_{C}\left(v\right)=\frac{1}{\sum_{u\neq v}d\left(v,u\right)} \end{array}$$ (12)

where *d*(*v*, *u*) denotes the distance between nodes *v* and *u*. Some researchers have proposed other definitions of closeness to measure the locations of nodes [25, 26].

*PageRank* [27] evaluates the status of nodes in the random walking process in networks, which is also a core algorithm in the many search engines.

$$\begin{array}{c} PR\left(v\right)=d\sum_{u\in \Gamma (v)}\frac{1}{k_{u}^{out}}PR\left(u\right)+\left(1-d\right)\frac{1}{N} \end{array}$$ (13)

where *N* denotes the total number of nodes, Γ(*v*) denotes the set of neighbors of *v*, $k_{u}^{out}$ denotes the out-degree of node *u*, and *d* is a dumping factor. In real implementations, we set *d* = 0.85.

*Coreness* [28] is a well-established centrality index that focuses on the structure of networks. Kitsak *et al.* found that the most efficient spreaders are those located within the core of the network as identified by the k-shell decomposition analysis. The decomposition runs in an iterative way. Nodes are assigned to *k* shells according to their remaining degrees, which are obtained by the successive pruning of nodes with degrees smaller than *k*_*s*_. However, the performance of k-shell decomposition is not stable, and many studies have sought to enhance its effectiveness [29–31]. Recently, Liu *et al.* [32] analyzed the structure of core-like groups in networks, and improved the accuracy of the k-shell decomposition by filtering out the redundant links. In Ref [33], Lü *et al.* discovered an important relation among degree, H-index and coreness. By constructing a suitable operator, they proved that degree, H-index and coreness were the initial, intermediate and steady states of a special sequences, respectively.

### Effectiveness

We use the SIR model to compare the effectiveness of *GeneralizedDegreeDiscount* with *DegreeDistance*, *SingleDiscount*, *DegreeDiscount* and several centrality-based methods discussed before. In each implementation, a fraction of the nodes is selected as spreaders, and the information spreads according to the SIR process described above. The spreading influence is used to measure the effectiveness of the methods. For each method, the SIR process is repeated many times to ensure the stability of the results.

**Fig 3:**
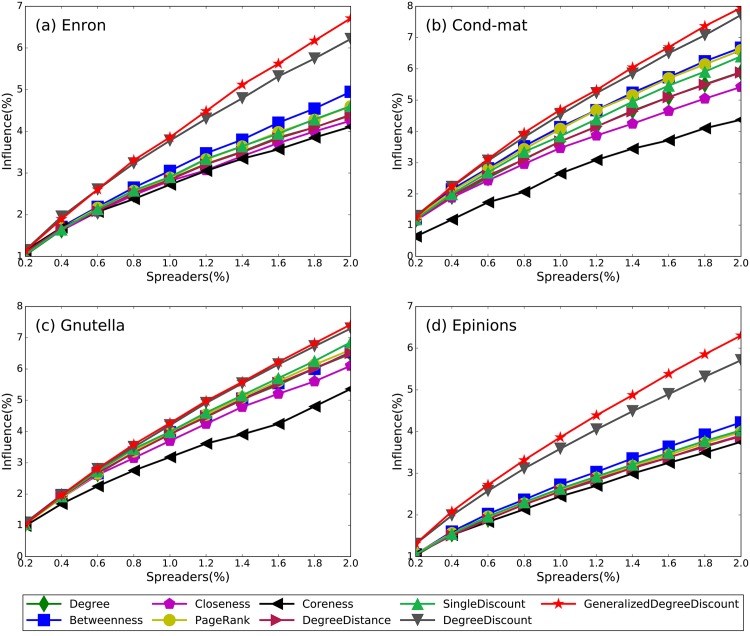
The spreading influence of nine methods on four networks with different fractions of spreaders. The parameters are *λ* = 1.1, *q* = 1/〈*k*〉 for all networks, and all results are obtained by averaging over 200 implementations of the SIR model.

Fig 3 shows the numerical results of nine methods on four networks. The proposed *GeneralizedDegreeDiscount* outperforms all other methods on all four networks for almost all fraction of spreaders. Especially, as the fraction of spreaders increases, our method shows better and better performance. The only exception is in Enron network, when the fraction of spreaders is small, the performance of *GeneralizedDegreeDiscount* is slightly worse than *DegreeDiscount*. Compared with *DegreeDiscount*, the performance of *GeneralizedDegreeDiscount* is consistently better. One promising phenomenon observed in our method is that as the fraction of spreaders becomes larger, the performance differences becomes more significant. Numerical results confirm that *GeneralizedDegreeDiscount* is indeed a effective extension of *DegreeDiscount*. In all networks, *Coreness* and *Closeness* perform the worst among all methods. In Ref [31], Liu *et al.* found that nodes in high shells may not be influential because of the existence of core-like groups: groups of nodes that link very locally within themselves. For nodes in the core-like groups, the *Coreness* cannot reflect their location importance in the network, which reduces the accuracy of the k-shell decomposition process. Moreover, if nodes in the highest shell tend to links with one another, their influence areas may overlap significantly. Obviously, selecting those nodes as spreaders may cause a large fraction of the network to overlooked. The situation for *Closeness* is similar: nodes with high closeness values often distribute closely with one another.

**Fig 4:**
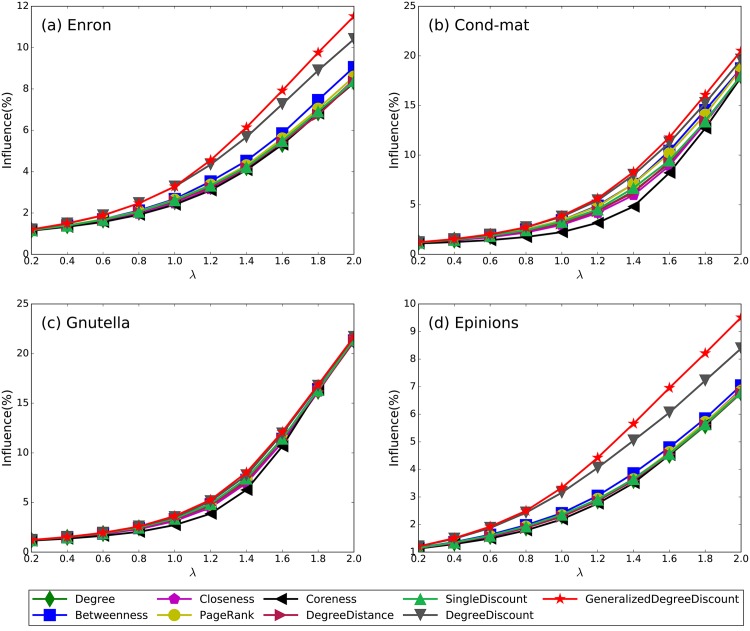
The spreading influence of nine methods on four networks under different effective spreading rates. The parameters are *λ* = 1.1, *q* = 1/〈*k*〉 for all networks, and all results are obtained by averaging over 200 implementations of the SIR model.

In addition, we test the validity of our method with different effective spreading rates. We fix the fraction of spreaders to be 1% of the scale of the networks and vary the effective spreading rate *λ*. The results are shown in Fig 4. As in the previous experiments, the proposed *GeneralizedDegreeDiscount* shows a clear advantage in maximizing the spreading influence over all networks under various effective spreading rates. Even if the effective spreading rates are below 1.0 in four networks, *GeneralizedDegreeDiscount* performs better than other methods.

**Fig 5:**
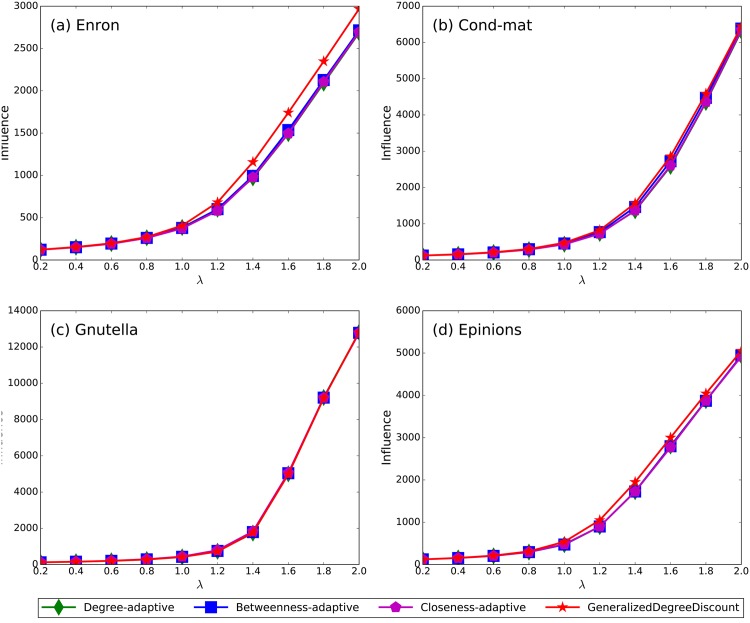
The spreading influence of GeneralizedDegreeDiscount and three adaptive centrality-based methods under different effective spreading rates. The numbers of spreaders are 100 in all networks, and the results are obtained by averaging over 200 implementations of the SIR model.

Obviously, *GeneralizedDegreeDiscount* is an adaptive method which recalculates the *gdd*_*v*_ during each step of the spreaders selection processes, while the centrality-based benchmark methods are not. In this part, more comparisons are done among our methods and adaptive versions of *Degree*, *Betweenness* and *Closeness*. To make them adaptive, a simple node-removing process is conducted: in each iteration, the node with the maximum centrality is selected as a spreader, and then we remove it from the network and recalculates the new centrality. The whole process ends until all the spreaders are selected. In fact, the adaptive version of *Degree* is the same as *SingleDiscount*. Fig 5 shows the numerical results. Unlike the previous results, when considering the top spreaders with low effective rate, our *GeneralizedDegreeDiscount* does not performs well. Especially in Gnutella network, the performance of *GeneralizedDegreeDiscount* is worse than *Betweenness-adaptive* and *Closeness-adaptive*. As the clustering coefficient of Gnutella is so small, the spreaders selection process in the early iteration of *GeneralizedDegreeDiscount* is just similar to *Degree*, which may limit the performance of our method. In Figs 3 and 4, it can also be seen that the performance differences between *GeneralizedDegreeDiscount* and other methods are not so remarkable under small number of spreaders and low effective spreading rate. How to identify multiple influential spreaders in networks with low clustering coefficients is a challenging problem, and we leave it in the future.

### Relations with other methods

In this subsection, we perform numerical comparisons among four degree-based methods: *Degree*, *SingleDiscount*, *DegreeDiscount* and *GeneralizedDegreeDiscount*. Though *DegreeDistance* is also a degree-based method, we do not consider it because there is no clear formulation to describe this method. The mathematical formulations of the four are listed below.

- *Degree*
  $$\begin{array}{c} d_{v}=d_{v} \end{array}$$
- *SingleDiscount*
  $$\begin{array}{c} sd_{v}=d_{v}-t_{v} \end{array}$$
- *DegreeDiscount*
  $$\begin{array}{c} dd_{v}=d_{v}-2t_{v}-\left(d_{v}-t_{v}\right)t_{v}p \end{array}$$
- *GeneralizedDegreeDiscount*
  $$\begin{array}{c} gdd_{v}=d_{v}-2t_{v}-\left(d_{v}-t_{v}\right)t_{v}p+\frac{1}{2}t_{v}\left(t_{v}-1\right)p-\sum_{d_{v}-t_{v}}t_{w}p \end{array}$$

**Fig 6:**
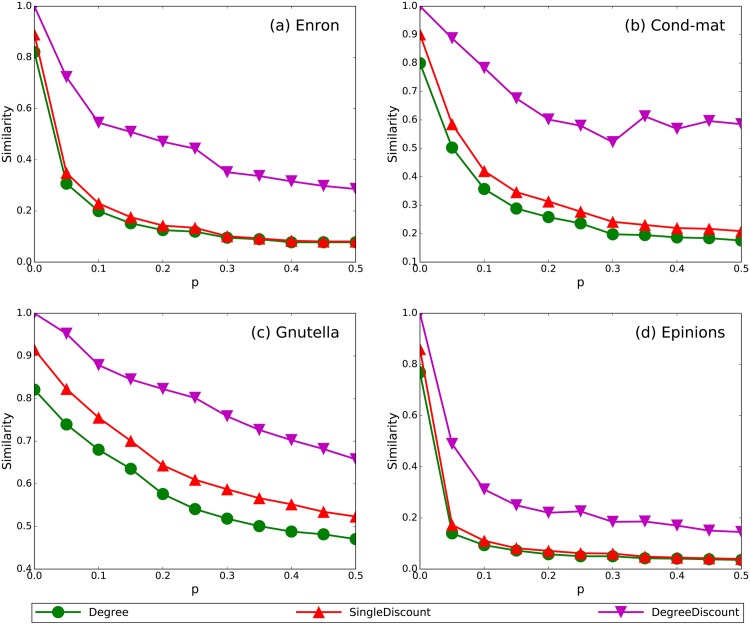
The similarities between *GeneralizedDegreeDiscount* and *Degree*, *SingleDiscount*, *DegreeDiscount*. The fraction of spreaders is 1%.

Obviously, the complexity of these methods increases one by one. These formulations indicate that *DegreeDiscount* has more terms in common with *GeneralizedDegreeDiscount* than the other two methods. In an extreme case, when *p* = 0, *GeneralizedDegreeDiscount* is exact the same as *DegreeDiscount*. To better clarify the difference between the methods, we set the fraction of spreaders to be 1% and calculate the similarity(the fraction of commonly selected spreaders) between *GeneralizedDegreeDiscount* and other methods. Fig 6 shows the results for the four networks. In all the networks, *GeneralizedDegreeDiscount* shows the best similarity with *DegreeDiscount*, normal similarity with *SingleDiscount*, and the worst similarity with *Degree*.

## Conclusion

In this paper, we propose a novel degree-based heuristic, *GeneralizedDegreeDiscount*, which selects multiple spreaders and maximizes their spreading influence. In our method, when evaluating the potential influence of a candidate node *v*, the way in which its neighbors are treated depends on whether it has been selected as a spreader or not. Taking both of the situations into consideration, *GeneralizedDegreeDiscount* uses a heuristic scheme to evaluate the potential influence of all individuals in the network.

We analyze the computational complexity of our method and show that it is just linearly correlated with the network scale. Then, the performance of our method is evaluated in four real networks from different fields. Results show that our method outperforms several centrality-based methods and other heuristic methods in all cases, no matter how many spreaders we choose to select or what the effective spreading rate is.

The theoretical analysis about influence maximization problem is still lacking. Although it has long been proven that there is a strong connection between the spreading process and the percolation process [34, 35], few researches have discussed the relationship between influence maximization and percolation. Recently, Morone and Makse pointed out that the influence maximization problem could be mapped onto optimal percolation problem in random networks [36], which might shed light on a new trend of future researches [37, 38]. Besides, we have witnessed the rapid development of theories and methods for temporal networks [39, 40]. Further researches on the influence maximization problem in temporal networks may also be a promising direction [41, 42].

## Supporting Information

S1 File Enron. Data of Enron email communication network.
(RAR)

S2 File Cond-mat. Data of Cond-mat collaboration network.
(RAR)

S3 File Gnutella. Data of Gnutella peer-to-peer network.
(RAR)

S4 File Epinions. Data of Epinions social network.
(RAR)
